# Prognostic Significance of High-Sensitivity Troponin-T and Hematological Biomarkers in Spontaneous Intracranial Hemorrhage Patients Undergoing Surgery

**DOI:** 10.3390/diagnostics15172274

**Published:** 2025-09-08

**Authors:** Akın Öztürk, Suna Dilbaz, Kadir Çakaroğlu, Abdurrahim Tekin, Engin Can, Evren Sönmez, Lokman Ayhan, Enes Özlük, Nuri Serdar Baş, Serdar Çevik

**Affiliations:** 1 Department of Neurosurgery, İstanbul Kanuni Sultan Süleyman Training and Research Hospital, University of Health Sciences, İstanbul 34303, Türkiye; akozturk@gmail.com (A.Ö.); sunadilbaz@gmail.com (S.D.); kcakaroglu@gmail.com (K.Ç.); tekinabdurrahim@gmail.com (A.T.); ecan281@gmail.com (E.C.); evren-sonmez@hotmail.com (E.S.); op.dr.lokmanayhan@gmail.com (L.A.); nuriserdarbas@gmail.com (N.S.B.); 2 Department of Radiology, İstanbul Kanuni Sultan Süleyman Training and Research Hospital, University of Health Sciences, İstanbul 34303, Türkiye; enesozluk@yahoo.com

**Keywords:** spontaneous intracranial hemorrhage (sICH), high-sensitivity troponin-i (hs-cTn-I), prognostic indicators, hematological biomarkers, surgical outcomes

## Abstract

**Background/Objectives:** Spontaneous intracranial hemorrhage (sICH) is a life-threatening condition with high in-hospital mortality rates. Prognostic evaluation remains challenging, and biomarkers such as neutrophil-to-lymphocyte ratio (NLR), platelet-to-lymphocyte ratio (PLR), lymphocyte-to-monocyte ratio (LMR), systemic immune-inflammation index (SII), glucose-to-lymphocyte ratio (GLR), and high-sensitivity troponin-I (hs-cTn-I) have been studied for their potential prognostic significance. This study aimed to evaluate the role of hs-cTn-I, NLR, PLR, LMR, and hematoma volume in predicting prognosis in sICH patients. **Methods:** This retrospective study included 49 adult patients (>18 years) admitted between January 2021 and January 2024 with sICH and hematoma volume >30 mL. All patients underwent surgery within 24 h of admission. Laboratory data, including hs-cTn-I levels and hematological indices, were collected. The hematoma volume was measured using the ABC/2 method. Patients were divided into survival and mortality groups. Statistical analyses were performed using SPSS, with *p* < 0.05 considered significant. **Results:** Of the 49 patients, 24 (49%) died. Admission hs-cTn-I levels showed no significant difference between groups, but levels on days 7 and 30 were significantly higher in the mortality group (*p* < 0.001). ROC analysis revealed hs-cTn-I levels on day 30 had better prognostic performance (AUC: 0.89, cut-off: 46 ng/mL, sensitivity: 76%, specificity: 88%). The hematoma volume and admission hematological indices (NLR, PLR, LMR, SII, and GLR) were not significantly associated with prognosis. **Conclusions:** Elevated hs-cTn-I levels, particularly on days 7 and 30, were significant predictors of in-hospital mortality in sICH patients. While admission hematological indices and hematoma volume lacked prognostic value, hs-cTn-I may serve as a valuable biomarker for risk stratification in clinical practice. Further multicenter studies with larger cohorts and multivariate analyses are needed to validate these findings.

## 1. Introduction

Spontaneous intracranial hemorrhage (sICH) is a group of diseases that includes hypertensive cerebral hemorrhage and hemorrhage due to autovascular causes. It poses a serious threat to patients’ lives. Despite advancements in intensive care and surgical interventions, the prognosis of sICH remains poor, with limited reliable biomarkers to guide clinical decision making [1]. Mortality rates have steadily increased over the last decade, with reports indicating that 40% of stroke-related deaths are due to hemorrhagic strokes [2]. Given the life-threatening nature of this condition, there is an urgent need for non-invasive and affordable tests that can reliably identify individuals at higher risk of death and enable timely measures to prevent death.

Some inflammatory biomarkers, such as neutrophil-to-lymphocyte ratio (NLR), platelet-to-lymphocyte ratio (PLR), and lymphocyte-to-monocyte ratio (LMR), have been reported in previous studies to have potential prognostic significance in ICH patients and have been associated with adverse prognosis [3,4,5,6]. Systemic immune-inflammation index (SII) is a predictor of adverse hospital discharge [7], while systemic inflammation response index (SIRI) is a predictor of 1-month mortality in ICH [8]. In addition, glucose-to-lymphocyte ratio (GLR), a composite measurement encompassing both glucose levels and systemic inflammation, may serve as a valuable indicator, offering a new foundation and guiding criteria for the clinical management of individuals with severe cerebral hemorrhage. Due to the high short-term mortality rate in spontaneous intracerebral hemorrhage (ICH) patients, research is still ongoing to improve prognostic evaluation for these cases.

Serum cardiac troponin has emerged in recent years as a novel test closely linked to vascular events [9]. Moreover, elevated troponin levels have been found to be associated with poor outcomes and mortality in stroke patients [10,11]. In patients with spontaneous intracerebral hemorrhage (sICH), elevated high-sensitivity cardiac troponin I (hs-cTn-I) levels after admission are linked to secondary cardiac stress or neurogenic injury. Lesch H. et al. found that this increase is primarily due to stroke-induced heart injury, mediated by the brain-heart axis, rather than isolated myocardial infarction [12]. This mechanism involves excessive sympathetic activation and catecholamine release following brain injury, which harms heart muscle cells through calcium overload, mitochondrial dysfunction, and changes such as contraction band necrosis [13].

Many studies in the literature have investigated the effect of cardiac troponin levels on determining prognosis or in-hospital mortality rates in ICH patients [14,15]. There are publications reporting that high troponin I values, especially at the time of admission, are associated with in-hospital mortality and poor prognosis. In addition, recent studies on troponin levels have shown that peak values of cTnI in serum, rather than admission levels, reflect poor outcomes, including death [14,16].

This study is designed to assess the prognostic significance of high-sensitivity troponin-I (hs-cTn-I) concentrations and hematologic biomarkers in individuals presenting with spontaneous intracranial hemorrhage (sICH). The research further aims to determine whether temporal changes in hs-cTn-I levels serve as predictors of in-hospital mortality. Specifically, the following research questions are examined: (1) Do hs-cTn-I levels, particularly those measured on days 7 and 30, provide effective prognostic information regarding mortality among sICH patients? (2) Do hematologic indices and hematoma volume hold prognostic relevance in this patient population?

## 2. Materials and Methods

### 2.1. The Patient Population

This study was conducted as a retrospective analysis of adults (>18 years old) who were admitted to our hospital’s emergency department between January 2021 and January 2024 and diagnosed with spontaneous intracranial hemorrhage (sICH) upon consultation with our clinic and underwent surgery with an intracranial hematoma volume of >30 mL on CT scans. After obtaining ethics committee approval (University of Health Sciences, Kanuni Sultan Süleyman Training and Research Hospital, Ethics Committee, Decision Number: KAEK/2024.05.99, Date: 24 May 2024), patient data were accessed from the hospital automation system. Exclusion criteria were as follows: Patients with secondary causes of intracranial hemorrhage (traumatic, arterio-venous malformation, aneurysm, tumor, infections, hemorrhagic transformation of ischemic stroke), patients having coagulopathy, as well as patients using heparin, low molecular weight heparin, glycoprotein IIb/IIIa antagonists, or oral anticoagulant therapy were not included in the study. Only patients requiring surgical intervention for hematomas larger than 30 mL were included in our study [17]. The study was designed to specifically focus on a defined subgroup of sICH patients requiring surgical management. Patients were divided into two groups as those who were discharged from the hospital and those who had mortality.

### 2.2. Evaluation of Laboratory Data

Demographic, clinical, and laboratory data of the patients were obtained from the hospital automation system. The calculated hematology indices included complete blood count (CBC), glucose, prothrombin time (PT), international normalized ratio (INR), activated partial thromboplastin time (APTT), and derived inflammatory indices; neutrophil-to-lymphocyte ratio (NLR), lymphocyte-to-monocyte ratio (LMR), platelet-to-lymphocyte ratio (PLR), as well as SII [NEU × PLT/(lymphocytes (LYM) × 1000)] and Glucose-lymphocyte ratio (GLR). Serum hs-cTn-I concentrations were measured by the Architect system (Abbott Diagnostics, Chicago, IL, USA) using the Abbott-Architect Troponin I assay. A standard 12-lead ECG was routinely recorded for all patients.

### 2.3. Measurement of Hematoma Volume

Diagnostic and imaging controls were performed with a Toshiba 256 scanner (Toshiba Aguilion Prime, Tochigi, Japan). Bleeding volume was measured using manual segmentation, including the entire visible lesion area. Analysis of the results was performed at the workstation by an independent radiologist blinded to the patient results.

To assess the volume of intracranial hematomas in ICH patients, the ABC/2 technique was used based on the initial CT scan. A represents the maximum linear length in centimeters (cm), B represents the maximum width in centimeters, and C represents the maximum depth in centimeters. Depth C was calculated by multiplying the number of slices in which the hematoma was observed by the slice thickness specified in the CT scan.

### 2.4. Statistics

Statistical analyses were performed using SPSS software (version 20.0; IBM Corp, 2012, Armonk, NY, USA). Categorical variables were expressed as numbers and percentages, and continuous variables were expressed as mean and standard deviation. A Student’s *t*-test was used to compare continuous variables, and categorical variables were analyzed using the chi-square or Fisher’s exact tests. ROC were used to calculate the cut-off values for variables associated with mortality. A *p*-value < 0.05 was considered statistically significant.

## 3. Results

Our study included a total of 49 individuals who underwent surgery due to sICH; 14 (28.6%) were female and 35 (71.4%) were male, and the mean age was 56.4 ± 11.99 (range 30–82). Twenty-four (49%) of the patients died. Of the patients who died, 6 were female and 18 were male. The mean GCS score of all patients at admission was 8.59 ± 3.51, the mean hemorrhage volume was 69.35 mL ± 37.69, and 32.7% were located in the deep zone. Table 1 shows the basic and clinical characteristics of the study population.

The mean hs-cTn-I levels of the patients in the survival group at admission were 17.89 ng/mL ± 36.18, the mean hs-cTn-I levels on the 3rd day were 50.69 ng/mL ± 151.39, the mean hs-cTn-I levels on the 7th day were 49.88 ng/mL ± 128.33, and the mean hs-cTn-I levels on the 30th day were 36.36 ng/mL ± 31.62, while the mean hs-cTn-I levels of the patients in the death group at admission were 18.88 ng/mL ± 19.15, the mean hs-cTn-I levels on the 3rd day were 61.95 ng/mL ± 50.44, the mean hs-cTn-I levels on the 7th day were 188.39 ng/mL ± 224.35, and the mean hs-cTn-I levels on the 30th day were 134.08 ng/mL ± 93.71 (*p* = 0.898, *p* = 0.728, *p* = 0.010, *p* < 0.001, respectively) There was no statistically significant difference between the groups with respect to complete blood count parameters and derived indices (Table 2).

In terms of hematoma volume, the mean hematoma volume at admission was 62.62 mL ± 25.03 in the survival group, while the mean hematoma volume was 76.37 mL ± 47.01 in the mortal group. Although the hematoma volume was higher in the mortality group, there was no statistically significant difference between the 2 groups (*p* = 0.235). There was no statistically significant difference between the two patient groups in terms of age, heart disease, previous stroke history, hyperlipidemia, DM, HT, aPTT, PT, INR, location of the hematoma, and admission GCS (Table 1).

**Figure 1:**
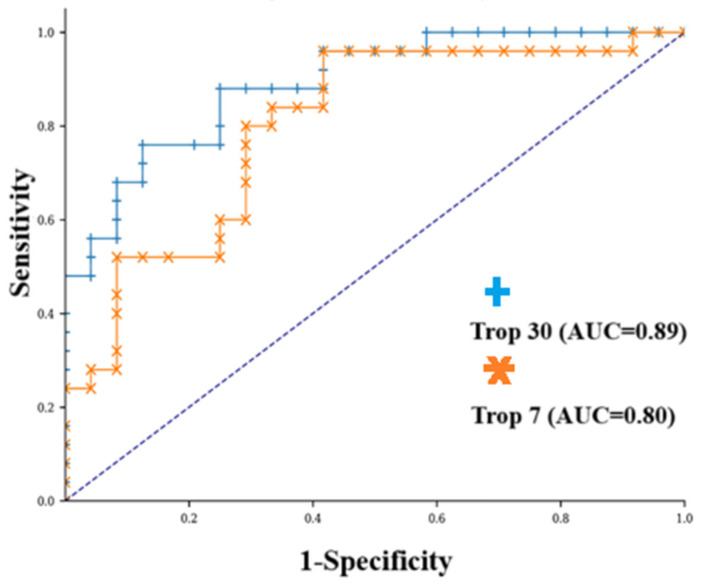
ROC curve of the seventh day hs-cTn-I to predict mortality for ICH. Youden index = 0.437 with associated criterion seven-day hs-cTn-I ≥ 19 ng/L. AUC = 0.80; 95% CI: 0.673–0.927; *p* < 0.001. Sensitivity: 52%. Specificity: 92%. Positive predictive value: 87%; negative predictive value: 64%. (orange plot) ROC curve of 30-day hs-cTn-I to predict mortality for ICH. Youden index = 0.635 with associated criterion 30th day hs-cTn-I ≥ 46 ng/L. AUC = 0.89; 95% CI: 0.803–0.977; *p* < 0.001. Sensitivity: 76%. Specificity: 88%. Positive predictive value: 86%; negative predictive value: 78% (blue plot).

ROC analysis was performed on the diagnostic results of hs-cTn-I levels on day 7 and day 30 according to the mortality variable of the study. The area under the receiver operating characteristic curve for hs-cTn-I levels on day 7 was 0.8 (95% CI: [0.673; 0.927]), the cut-off value was 19 ng/mL, sensitivity was 52% and specificity was 92%, PPV was 87%, and NPV was 64%. Hs-cTn-I levels on day 30 showed a better performance in predicting mortality with AUC 0.89 (95% CI: [0.803; 0.977]). The cut-off value of 46 ng/mL on day 30 provided sensitivity of 76% and specificity of 88%, PPV was 86%, and NPV was 78% (Figure 1).

## 4. Discussion

In this observational sICH cohort, we explored the prognostic value of admission NLR, LMR, GLR, SII, SIRI, and hs-cTn-I as biomarkers. sICH is linked to high mortality and morbidity. Prognosis in sICH patients can be predicted by factors like age, sex, hypertension history, anticoagulant use, GCS score, and hematoma volume. Additionally, certain biochemical biomarkers aim to aid risk stratification and ease care burden. Our study assessed the impact of these parameters on early prognosis in sICH patients. Results show that hs-cTn-I levels at diagnosis and on days 7, and 30 are significant and tied to prognosis. Admission hematological parameters, derived indices, and hematoma volume were not prognostically significant. In the literature, elevated hs-cTn-I levels at hospital admission are generally associated with poor prognosis. However, in our study, baseline hs-cTn-I levels were found to have no prognostic value. This could be attributed to several factors. All patients in the study underwent surgical intervention within the first 24 h of admission. Early intervention may have reduced intracranial pressure and cardiac stress, potentially limiting the prognostic impact of baseline hs-cTn-I levels. Additionally, hs-cTn-I levels measured at admission may not fully reflect the delayed cardiac effects of sICH. Research has indicated that troponin measurements performed later, such as 72 h after admission, correlate more closely with patient outcomes, as they represent ongoing systemic and cardiac stress rather than the immediate response to hemorrhage [18].

The reasons for elevated hs-cTn-I levels in sICH patients remain unclear. Cardiac troponin is a highly sensitive and specific marker of myocardial injury [19]. Widely used as a key biomarker in MI diagnosis, hs-cTn-I has recently been considered for assessing prognosis in various other conditions. Xu et al. observed that elevated cardiac troponin levels, after adjusting for known risk factors such as age, GCS score, and admission systolic blood pressure, were associated with larger hematoma volume in follow-up, as shown in bivariate correlation and linear regression [20]. Additionally, Xu et al. reported that elevated troponin levels were linked not only to larger hematoma volume but also to the frequency of cerebral herniation and deep hematoma localization [20]. In our study, although the mortality group had a higher mean hematoma volume compared to the survival group, the difference was not statistically significant. We hypothesize that this may be due to early surgical intervention within the first 24 h, which evacuated the hematoma and reduced intracranial pressure in all patients. In our study, only patients who underwent surgical intervention within the first 24 h were included. This selection criterion may have altered the natural progression of prognostic factors by limiting the impact of early surgical intervention on hematoma volume and inflammatory response. Consequently, it is difficult to compare our results with those of patients who did not undergo surgery or received surgery at a later stage. Therefore, our findings can only be generalized to patients who underwent early surgical intervention. By focusing exclusively on a subgroup of sICH patients deemed eligible for surgery, this criterion introduces a potential selection bias.

While our study did not find a significant association between admission hs-cTn-I levels and prognosis, previous studies have reported prognostic value for troponin at admission in intracerebral hemorrhage (ICH) patients. For instance, He et al. demonstrated that elevated cardiac troponin I levels at admission were associated with increased mortality in ICH patients [14]. Similarly, Ulger et al. found that admission hs-cTn-I levels above 26 ng/mL were linked to a 2.586-fold increase in mortality risk [15]. These findings suggest that early troponin elevation may reflect acute myocardial injury or systemic stress caused by the initial hemorrhagic event. However, despite the potential of cardiac troponin as a prognostic marker, the scientific literature provides conflicting findings regarding its association with the risk of all-cause mortality in patients with acute stroke [21,22,23,24,25]. This may be due to the evaluation of hs-cTn-I levels at the time of presentation or within the first 24 h. For this reason, Gueette et al. associated troponin levels > 22 ng/L at the 72nd h after the onset of symptoms in patients with aneurysmal subarachnoid hemorrhage with poor prognosis [26]. Gerner et al., in their study evaluating patients who were categorized according to the highest troponin I level during hospitalization, reported that troponin I elevations during hospitalization are frequently seen in ICH patients and are independently associated with functional outcomes at 3 and 12 months [16]. In contrast, our study highlights the importance of monitoring troponin levels over time, particularly on days 7 and 30, as delayed elevations showed stronger prognostic significance. In our study, it was detected that the two groups were not significantly different in terms of hs-cTn-I levels taken at the time of initial admission. However, there were significant differences in hs-cT-I levels on days 7 and 30. It was observed that hs-cT-I levels peaked especially on day 7. While the mean hs-cTn-I levels of the mortality group on days 7 and 30 were 188.4 ng/L and 134.1 ng/L (respectively), the mean hs-cTn-I levels of the survival group were 49.9 ng/L and 36.4 ng/L. (*p* = 0.010, *p* < 0.001, respectively). In addition, Hs-cTn-I levels on day 30 showed a better performance in predicting mortality with AUC 0.89 (95% CI: [0.803; 0.977]). The cut-off value of 46 ng/mL on day 30 provided a sensitivity of 76% and specificity of 88%, PPV was 86%, and NPV was 78%. In this study, the delayed troponin elevation observed, particularly on days 7 and 30, could be attributed to several mechanisms in the context of neurosurgical intervention. One possible explanation is the stress response triggered by intracranial hemorrhage and subsequent surgery. Acute cerebral lesions and elevated intracranial pressure may stimulate the sympathoadrenal system, leading to catecholamine release and myocardial injury, as previously suggested [12,13]. Furthermore, the surgical intervention itself may contribute to systemic inflammation and oxidative stress, which may exacerbate myocardial strain and elevate troponin levels over time. Another possible mechanism is the gradual progression of secondary brain injury, including cerebral edema and inflammation, which may indirectly affect cardiac function via neurogenic pathways. Furthermore, the delayed troponin elevation may reflect ongoing myocardial injury due to persistent systemic stress or hemodynamic instability after surgery. These mechanisms highlight the complex interaction between neurosurgical intervention, systemic inflammation, and cardiac biomarkers and warrant further investigation to better understand their prognostic implications.

Our study also revealed that initial serum levels of NLR, GLR, LMR, SII, and SIRI at admission were not associated with prognosis in patients with sICH. ICH induces systemic and peripheral inflammation, increasing circulating white blood cells (WBCs) and attracting specific molecules to the affected area, thereby exacerbating local damage [27]. Subsequently, pro-inflammatory cytokines and chemokines are released, promoting peripheral inflammatory infiltration and contributing to secondary injury [28,29]. The neutrophil-to-lymphocyte ratio (NLR) is considered a useful marker of systemic inflammation and reflects the balance between systemic inflammatory and immune responses [30]. In a study by Wang et al., which evaluated 224 patients, mortality was significantly higher in patients with an NLR level ≥ 7.35 at admission compared to those with an NLR < 7.35 (31.6% vs. 4.8%) [31]. However, that study also reported that a hematoma volume greater than 30 mL and a low GCS were additional prognostic factors. In a recent study by Radu et al., NLR levels measured 72 h after admission were identified as independent predictors of in-hospital mortality in ICH patients, and they suggested that NLR could be a widely applicable biomarker in clinical practice for identifying patients at high risk of in-hospital death [32]. Notably, this study excluded patients who underwent surgery. Many studies in the literature have linked parameters such as high leukocyte, neutrophil, and monocyte counts, low lymphocyte counts, and elevated NLR at admission with large hematoma volumes and poor prognosis [33]. In our study, none of the hematological parameters or their derived indices showed a statistically significant association with prognosis. In both of our patient groups, all individuals had a hematoma volume >30 mL at admission. We believe that the uniformly high hematoma volume at admission may explain the lack of association. Furthermore, we did not assess follow-up hematological parameters, as all patients underwent surgery within the first 24 h—a factor that is widely acknowledged to significantly alter these parameters.

This study has several limitations. First, the single-center, retrospective nature of this study limits the generalizability of the results. Future multicenter and prospective studies are necessary to confirm our findings. Second, the number of patients in our study was limited, especially for multivariate analyses. A larger sample size would provide stronger statistical results. Third, our study only evaluated 30-day outcomes. A longer follow-up may be useful to assess the long-term prognostic value of hs-cTn-I. Fourth, our study only evaluated hematological parameters at baseline. Examining changes in these parameters during follow-up would provide a more comprehensive assessment. Fifth, the fact that all patients received surgery within the first 24 h may affect the prognostic value of hematoma volume and hematological parameters. To better understand the impact of this factor, studies including patients who did not undergo surgery could be conducted.

## 5. Conclusions

Routine monitoring of hs-cTn-I levels on days 7 and 30 post-admission may improve prognostic accuracy and guide clinical decision-making in sICH patients undergoing surgery. Hs-cTn-I could serve as a cost-effective and widely available biomarker for risk stratification, aiding clinicians in identifying high-risk patients and tailoring postoperative care. However, the lack of multivariate analysis to adjust for confounding factors leaves the independent prognostic value of hs-cTn-I unclear. Larger cohort, multicenter, prospective studies and comprehensive statistical analyses are needed to confirm these findings and establish hs-cTn-I as a reliable biomarker for risk stratification.

## Figures and Tables

**Table 1:** Demographic, clinical, and imaging characteristics of the study population.

	Overall (*n* = 49)	Alive at Discharge (*n* = 25)	Mortality (*n* = 24)	*p* Value
Gender				0.588
male, *n* (%)	35 (71.4%)	17 (68%)	18 (75%)	
female, *n* (%)	14 (28.6%)	8 (32%)	6 (25%)	
Age, (years) mean ± SD	56.37 ± 11.99	54.2 ± 9.14	58.63 ± 14.23	0.200
GCS, mean ± SD	8.59 ± 3.51	8.64 ± 4.02	8.54 ± 2.96	0.923
Hypertension, *n* (%)	36 (73.5%)	16 (64%)	20 (83.3%)	0.125
Diabetes mellitus, *n* (%)	28 (57.2%)	12 (48%)	16 (66.7%)	0.187
Smoker, *n* (%)	33 (67.3%)	18 (72%)	15 (62.5%)	0.478
Heart disease, *n* (%)	9 (18.4%)	4 (16%)	5 (20.8%)	0.662
Previous Stroke History				
İschemic, *n* (%)	7 (14.3%)	4 (16%)	3 (12.5%)	0.726
Hemorrhagic, *n* (%)	1 (2.04%)	1 (4%)	1 (4.17%)	0.977
Hyperlipidemia, *n* (%)	13 (26.5%)	5 (25%)	8 (33.3%)	0.291
Prothrombin time, mean ± SD	15.35 ± 7.79	13.43 ± 3.16	15.69 ± 5.87	0.098
Activated partial thromboplastin time, mean ± SD	28.72 ± 8.87	27.19 ± 5.25	30.31 ± 11.41	0.222
International normalized ratio, mean ± SD	1.25 ± 0.65	1.09 ± 0.13	1.27 ± 0.55	0.109
WBC, (10^3^/μL)	15.46 ± 4.98	15.2 ± 4.21	15.75 ± 5.76	0.709
NEU, (10^3^/μL)	12.12 ± 4.37	11,77 ± 3.65	12.48 ± 5.08	0.578
LYM, (10^3^/μL)	2.16 ± 1.94	2.37 ± 2.24	1.95 ± 1.58	0.466
MON, (10^3^/μL)	0.62 ± 0.32	0.57 ± 0.28	0.68 ± 0.36	0.220
PLT, (10^3^/μL)	224.2 ± 75.83	220 ± 90	228.5 ± 59.26	0.698
Hematoma volume, cm^3^	69.35 ± 37.69	62.62 ± 25.03	76.37 ± 47.01	0.235
Hematoma location				0.921
Supratentorial labor	33 (67.3%)	17 (68%)	16 (66.7%)	
Supratentorial deep	16 (32.7%)	8 (32%)	8 (33.3%)	

**Table 2:** Baseline biomarkers and calculated indexes, and 1st, 3rd, 7th, and 30th day hs-cTn-I levels.

	Overall (*n* = 49)	Alive at Discharge (*n* = 25)	Mortality (*n* = 24)	*p* Value
WBC, (10^3^/μL)	15.46 ± 4.98	15.2 ± 4.21	15.75 ± 5.76	0.709
NEU, (10^3^/μL)	12.12 ± 4.37	11.77 ± 3.65	12.48 ± 5.08	0.578
LYM, (10^3^/μL)	2.16 ± 1.94	2.37 ± 2.24	1.95 ± 1.58	0.466
MON, (10^3^/μL)	0.62 ± 0.32	0.57 ± 0.28	0.68 ± 0.36	0.220
PLT, (10^3^/μL)	224.2 ± 75.83	220 ± 90	228.5 ± 59.26	0.698
SII	2.19 ± 1.74	2.07 ± 1.83	2.31 ± 1.67	0.639
SIRI	5.85 ± 5.91	4.89 ± 5.33	6.85 ± 6.42	0.250
NLR	9.95 ± 7.54	9.73 ± 8.53	10.18 ± 6.51	0.838
LMR	3.92 ± 3.63	4.68 ± 4.54	3.14 ± 2.15	0.140
GLR	8.12 ± 6.65	6.48 ± 4.98	9.82 ± 7.77	0.78
hs-cTn-I 1st day (ng/mL)	18.4 ± 28.82	17.9 ± 38.18	18.88 ± 19.15	0.897
hs-cTn-I 3rd day (ng/mL)	56.21 ± 112.75	50.69 ± 151.39	61.95 ± 50.44	0.728
hs-cTn-I 7th day (ng/mL)	117.72 ± 193	49.88 ± 128.33	188.39 ± 224.35	0.010
hs-cTn-I 30th day (ng/mL)	84.22 ± 84.52	36.36 ± 31.62	134.08 ± 93.71	<0.001

## Data Availability

All The original contributions presented in this study are included in the article. Further inquiries can be directed to the corresponding author.

## Abbreviations

sICH	Spontaneous intracranial hemorrhage
NLR	neutrophil-to-lymphocyte ratio
PLR	platelet-to-lymphocyte ratio
MLR	lympho-cyte-to-monocyte ratio
LMR	lympho-cyte-to-monocyte ratio
SII	systemic immune-inflammation index
GLR	glucose-to-lymphocyte ratio
Hs-cTn-I	high-sensitivity troponin-I
